# Functional anterior pituitary generated in self-organizing culture of human embryonic stem cells

**DOI:** 10.1038/ncomms10351

**Published:** 2016-01-14

**Authors:** Chikafumi Ozone, Hidetaka Suga, Mototsugu Eiraku, Taisuke Kadoshima, Shigenobu Yonemura, Nozomu Takata, Yutaka Oiso, Takashi Tsuji, Yoshiki Sasai

**Affiliations:** 1 Laboratory for Organogenesis and Neurogenesis, RIKEN Center for Developmental Biology, Kobe 650-0047, Japan; 2 Laboratory for Organ Regeneration, RIKEN Center for Developmental Biology, Kobe 650-0047, Japan; 3 Department of Endocrinology and Diabetes, Graduate School of Medicine, Nagoya University, Nagoya 466-8550, Japan; 4 Laboratory for In Vitro Histogenesis, RIKEN Center for Developmental Biology, Kobe 650-0047, Japan; 5 Electron Microscope Laboratory, RIKEN Center for Developmental Biology, Kobe 650-0047, Japan; 6 CREST, Japan Science and Technology Agency, Kobe 650-0047, Japan

## Abstract

Anterior pituitary is critical for endocrine systems. Its hormonal responses to positive and negative regulators are indispensable for homeostasis. For this reason, generating human anterior pituitary tissue that retains regulatory hormonal control *in vitro* is an important step for the development of cell transplantation therapy for pituitary diseases. Here we achieve this by recapitulating mouse pituitary development using human embryonic stem cells. We find that anterior pituitary self-forms *in vitro* following the co-induction of hypothalamic and oral ectoderm. The juxtaposition of these tissues facilitated the formation of pituitary placode, which subsequently differentiated into pituitary hormone-producing cells. They responded normally to both releasing and feedback signals. In addition, after transplantation into hypopituitary mice, the *in vitro*-generated corticotrophs rescued physical activity levels and survival of the hosts. Thus, we report a useful methodology for the production of regulator-responsive human pituitary tissue that may benefit future studies in regenerative medicine.

## Abstract (web summary)

It is difficult to generate functional human anterior pituitary tissues *in vitro*. Here, Ozone *et al.* generate human anterior pituitary from embryonic stem cells by recapitulating *in vivo* development, and demonstrate this tissue secretes hormones and rescues hypopituitarism when grafted into mice.

## Graphical abstract

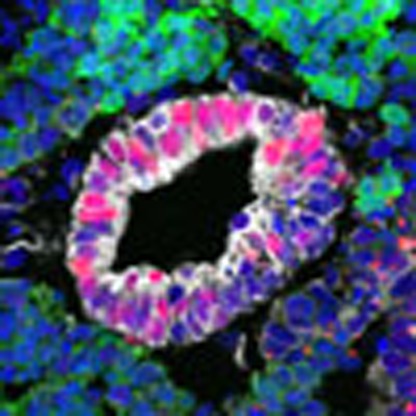

The anterior pituitary is a key endocrine centre for systemic hormones, secreting hormones such as adrenocorticotropic hormone (ACTH) and growth hormone (GH) that are critical for survival, homeostasis and growth^1^,^2^. Disorders of the pituitary can cause various maladies, some of which are life-threatening. In addition, although current hormone-replacement therapies can alleviate some of these conditions, exogenous hormone administration cannot recapitulate the natural and precise regulatory control of the endogenous endocrine system. For example, as for ACTH deficiency, once the hormone replacement is insufficient, the patient's life will be immediately in danger because of the adrenal failure. In case the replacement is excess, the patient will suffer from the side effects, such as obesity, diabetes mellitus, hypertension, hyperlipidaemia, osteoporosis and depression, in years to come. Furthermore, the adequate dose fluctuates both hourly and daily. For this reason, the ability to create human pituitary tissue, which can respond to the surrounding environment, amenable to effective curative therapies for pituitary dysfunction would be a huge advance for regenerative medicine. Towards this aim, Dincer *et al.*^3^ reported the generation of pituitary hormone-producing cells from human pluripotent stem cells, yet whether these cells were responsive (that is, could respond to their regulatory cues) was not determined. Furthermore, no report has been made for therapeutic ability of human pluripotent stem cell-derived pituitary tissues in animal transplantation so far.

In this study, we therefore endeavoured to utilize human embryonic stem cells (hESCs) to generate regulator-responsive anterior pituitary tissue *in vitro* capable of the *in vivo* treatment of hypopituitarism. To reach this goal, we develop the self-organizing culture of hESCs that enables pituitary primordium formation *in vitro* by recapitulating *in vivo* development. The hESC-derived pituitary progenitors differentiated into mature hormone-producing cells, such as corticotrophs and somatotrophs. They secreted ACTH and GH, respectively, in response to positive and negative regulatory signals. Furthermore, when we transplanted *in vitro*-generated corticotrophs *in vivo*, they improved activity levels and survival of hypopituitary mice. Thus, hESC-derived pituitary tissues shown here provide a platform for therapeutic application and disease modelling.

## Results

### Pituitary placode formation by recapitulating embryogenesis

**Figure 1:**
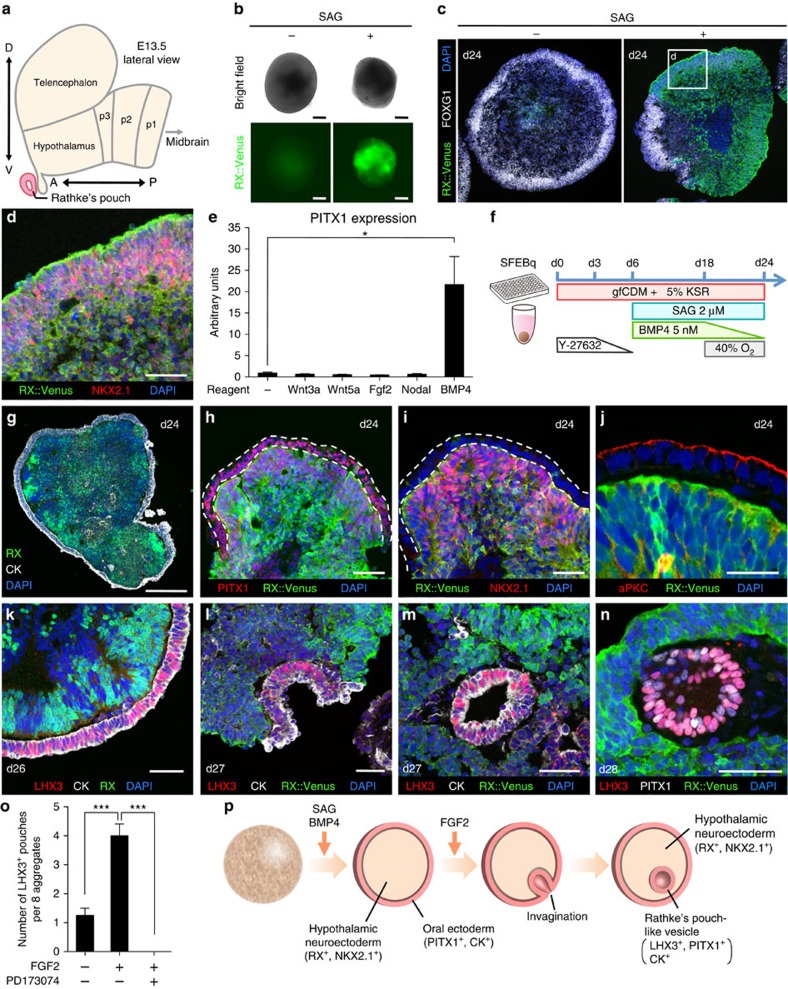
Generation of a Rathke's pouch-like structure in 3D culture of hESC aggregates. (**a**) Schematic (lateral view; modified from ref. 10) of the prosomeric domains in the E13.5 mouse forebrain. A, anterior; D, dorsal; P, posterior; p1–3, prosomere 1–3; V, ventral. (**b**) *RX*::Venus expression on day-24 aggregates with or without SAG treatment. Upper panels, bright-field view. (**c**) Immunostaining of day-24 aggregates with/without SAG treatment for *RX*::Venus (green) and FOXG1 (white). *RX*::Venus expression was induced by SAG treatment. d, day. (**d**) SAG-treated NE co-expressed *RX*::Venus (green) and the ventral marker NKX2.1 (red). (**e**) Increased PITX1 (oral ectoderm marker) expression in day-24 aggregates treated with 5 nM BMP4 (qPCR; *n*=3 experiments). Wnt3a (50 ng ml^−1^ final concentration), Wnt5a (50 ng ml^−1^), FGF2 (50 ng ml^−1^), Nodal (50 ng ml^−1^) and BMP4 (5 nM) were added to the culture medium during days 6–18. All of them are recombinant human proteins. (**f**) Culture protocol for pituitary placode induction. (**g**–**j**) Adjacent formation of oral ectoderm (pan-Cytokeratin^+^, PITX1^+^) and ventral hypothalamic NE (RX^+^, NKX2.1^+^) in 3D hESC culture. (**h**,**i**) Serial sections. CK, pan-Cytokeratin. (**j**) aPKC (the apical marker) immunostaining of day-24 aggregates. (**k**–**n**) Morphogenesis of Rathke's pouch-like structure *in vitro*. (**k**) Thickened oral ectoderm (Cytokeratin^+^; white) in aggregates expressed the pituitary placode marker LHX3 (red) on day 26. (**l**–**n**) Immunostaining of day 27–28 pouch vesicles and surrounding neural tissues for LHX3 (red; **l**–**n**), pan-Cytokeratin (white; **l**,**m**), PITX1 (white; **n**) and *RX*::Venus (green; **l**–**n**). (**o**) Increased the number of LHX3^+^ pouch vesicles per eight aggregates on day 27 by FGF2 treatment (*n*=4 experiments). PD173074 (FGF receptor inhibitor) suppressed the formation of the pouches (*n*=4 experiments). (**p**) Schematic of generation of Rathke's pouch-like structure in 3D culture of hESCs. Scale bars, 200 μm (**b**,**c**,**g**), 50 μm (**d**,**h**–**n**). The values shown on graphs represent the mean±s.e.m. *P<0.05, ***P<0.001 using one-way analysis of variance (ANOVA) with Dunnett's test (**e**,**o**).

Our approach was to recreate pituitary embryonic development within three-dimensional (3D) culture conditions. During early embryogenesis, the pituitary primordium (Rathke's pouch) emerges from the oral ectoderm under the inductive influences of overlying ventral hypothalamic neuroepithelia (NE)^4^,^5^,^6^,^7^,^8^,^9^ (Fig. 1a and Supplementary Fig. 1a). To mimic this process *in vitro*, we utilized a 3D culture method called SFEBq (serum-free floating culture of embryoid body-like aggregates with quick reaggregation)^10^ (Supplementary Fig. 1b). Indeed, previous studies^11^ using mouse ES cells (mESCs) showed that hypothalamic NE can be co-induced with non-neural ectoderm within the same SFEBq aggregates by high-density cell plating and optimized culture media. This method allowed functional pituitary placode tissues to self-form from non-neural oral ectoderm via local interactions with hypothalamic NE.

To generate pituitary tissues from human pluripotent stem cells, we sought to determine which SFEBq culture conditions would promote the generation of pituitary placode tissues. In particular, we sought a culture method that would facilitate the co-induction of two key tissues, ventral hypothalamus and non-neural ectoderm, which we hypothesized would then interact and promote the development of the pituitary placode.

First, we optimized the differentiation conditions for ventral hypothalamic NE (RX^+^/NKX2.1^+^) in hESC aggregates. We previously showed that *in vitro* murine hypothalamic differentiation is best induced with growth factor-free chemically defined medium (gfCDM)^12^. On the basis of this idea, we chose gfCDM supplemented with Knockout Serum Replacement (KSR) and the Rho kinase (ROCK) inhibitor Y-27632 (suppressing hESC-dissociation-induced apoptosis)^13^ to start neural differentiation using SFEBq culture (Supplementary Fig. 1b). For these experiments, we used an *RX*::Venus reporter hESC line^14^, allowing us to monitor hypothalamic differentiation in real time. Using this initial gfCDM/KSR/Y-27632 culture media, we found that, by day 24, *RX*::Venus signals were not yet visible (Fig. 1b; SAG (−) panels), and immunostaining analysis showed that the NE in these aggregates expressed FOXG1, TUJ and NCAD, suggesting that telencephalic, not hypothalamic, differentiation was induced (Fig. 1c and Supplementary Fig. 1c–e; SAG (−) panel). Thus, we next promoted a more ventral positional identity by augmenting hedgehog signalling with smoothened agonist SAG^15^. Indeed, we found that the addition of SAG (2 μM, days 6–24) robustly induced *RX*::Venus signals (Fig. 1b,c), and *RX*::Venus^+^ tissues expressed the ventral hypothalamic marker NKX2.1 (Fig. 1d).

We next sought to establish which conditions would induce non-neural ectoderm formation in the SAG-treated *RX*::Venus^+^ aggregates. Previous experiments^11^ using mESCs showed that non-neural ectodermal differentiation is promoted by high cell-density conditions, that is, increasing the plating-cell number to 10,000 cells per well. However, we found that these conditions did not promote non-neural ectoderm differentiation from hESCs. Therefore, we tried various other culture conditions that would promote non-neural ectoderm differentiation. Among these, we found that early exposure to bone morphogenetic protein 4 (BMP4; known to favour non-neural ectoderm differentiation at the cost of neural differentiation)^11^,^16^,^17^,^18^ was effective (Fig. 1e). We found that adding 5 nM BMP4 (final concentration) to the culture medium from day 6 (Fig. 1f) led to the generation of pan-Cytokeratin^+^ non-neural ectoderm (also ECAD^+^; Fig. 1g and Supplementary Fig. 1f,g). This treatment formed superficial layers surrounding the ventral hypothalamic NE (RX^+^/NKX2.1^+^; Fig. 1g–j and Supplementary Fig. 1f–k; day 24). Notably, the surface ectoderm also expressed the early pituitary/oral ectodermal marker PITX1 (Fig. 1h), and the apical marker aPKC was localized to the outer side of the ectoderm, identical to the mouse development pattern (Fig. 1j and Supplementary Fig. 1l). In this culture condition, ventral hypothalamic neural tissues co-existed intact, dispite BMP4 treatment, a property that is unlike that seen in mESC culture^11^ (Fig. 1i).

By days 26–28, we observed that parts of pan-Cytokeratin^+^ oral ectoderm were thickened and expressed the early pituitary marker LHX3 (also called LIM3)^19^,^20^ (Fig. 1k), a portion that was often curved (Fig. 1l), reminiscent of invagination of Rathke's pouch (Supplementary Fig. 1a) and formed a hollowed vesicle (Fig. 1m,n). However, these LHX3^+^ Rathke's pouch-like vesicles were relatively rare (1.3±0.3 vesicles per eight aggregates; mean±s.e.m., *n*=4; Fig. 1o and Supplementary Fig. 1m). Thus, to further promote the formation of LHX3^+^ vesicles, we examined the effect of fibroblast growth factor (FGF) signalling, which had been implicated in promoting early pituitary development^6^,^7^,^8^,^21^. When FGF2 was added to the medium from day 15 (20 ng ml^−1^ until day 27), LHX3^+^ pouch-like vesicles were observed more frequently than in non-FGF2 conditions (4.0±0.4 vesicles per eight aggregates; mean±s.e.m., *n*=4) and no vesicles were found when the FGF receptor inhibitor PD173074 was added to the FGF2-treated culture (Fig. 1o). FGF signals such as FGF8 and FGF10 are required in mouse Rathke's pouch development^6^,^7^,^8^,^21^. FGF2 has the ability to bind both FGF8 and FGF10 receptors^22^,^23^. For these reasons, we considered that exogenous FGF2 promoted the formation of Rathke's pouch-like structures *in vitro*. FGF8 and FGF10 expression levels were gradually elevated in the aggregates during days 15–27 (Supplementary Fig. 2a,b). Both PD173074 and SU5402 (FGF receptor inhibitors) repressed the expression of *PITX1* and *LHX3* in the hESC culture (Supplementary Fig. 2c,d). These data indicate that endogenous FGF signals have a role in *PITX1* and *LHX3* induction, at least in part. We also found that SAG increased GLI1 expression levels in hESC aggregates (Supplementary Fig. 2e,f). GLI inhibitors HPI-1 and GANT61 suppressed GLI1 expression and subsequent *PITX1* and *LHX3* expression in hESC culture (Supplementary Fig. 2g,h), suggesting that hedgehog–GLI signalling is required for pituitary differentiation.

Thus, with the combined application of hedgehog and BMP4 signals, hESCs differentiate into both oral ectoderm and the hypothalamic NE within the same aggregate. When these aggregates were exposed to further FGF signals, the surface ectoderm induced the formation of Rathke's pouch-like structures (Fig. 1p). In contrast, PITX1^+^ oral ectoderm tissue alone (lacking RX^+^ hypothalamic NE) did not exhibit LHX3^+^ pituitary placode formation even on day 53 (Supplementary Fig. 2i,j), suggesting the possibility that adjacent ectodermal layers may be required for *LHX3* induction. We speculate that the aggregates (PITX1^+^, LHX3^−^ and RX^−^) have the ability to differentiate into other oral ectodermal tissue, such as oral and nasal epithelia^24^.

### Generation of pituitary hormone-producing cells from hESCs

**Figure 2:**
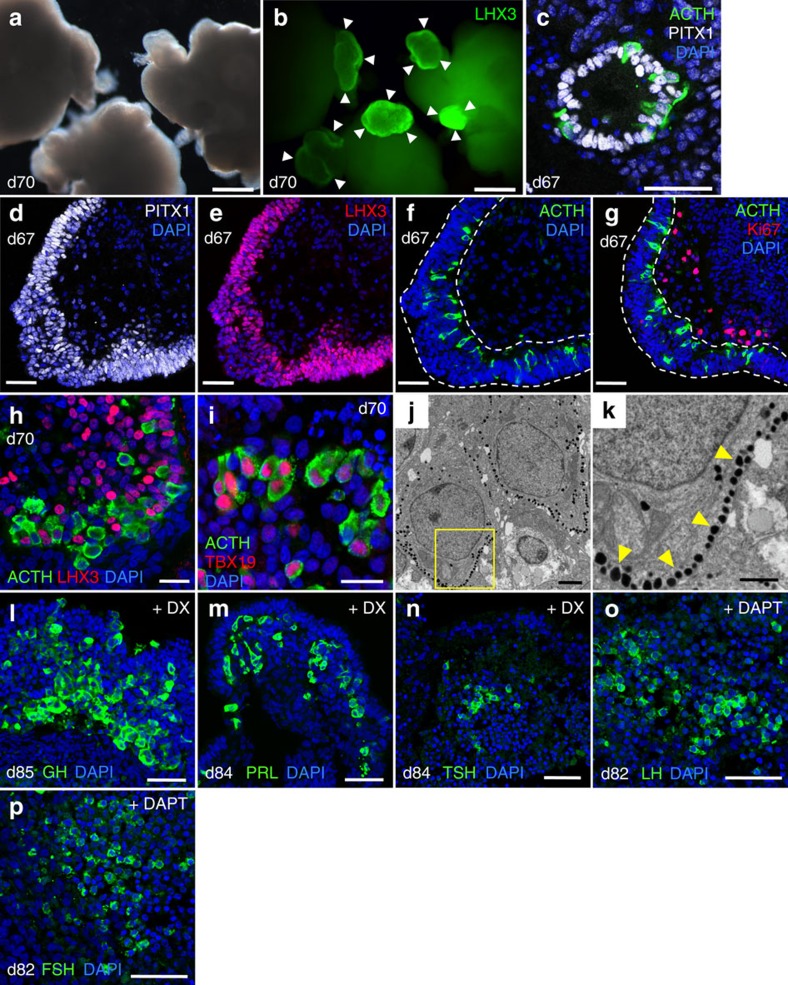
Differentiation and maturation of hESC-derived pituitary progenitors into multiple hormone-producing tissues. (**a**,**b**) Bright-field (**a**) and fluorescence (**b**) views of day-70 hESC aggregates stained for LHX3 (green). Arrowheads, LHX3^+^ epithelia. (**c**) PITX1^+^ pouch-forming ectoderm (white) generated ACTH^+^ (green) cells in day-67 aggregates. (**d**–**g**) Immunostaining of non-pouch-forming oral ectoderm for PITX1 (white; **d**), LHX3 (red; **e**), ACTH (green; **f**) and the proliferation marker Ki67 (red; **g**). (**h**) ACTH^+^ cells (green) did not express LHX3 (pituitary progenitor marker) on day 70. (**i**) Induced ACTH^+^ cells (green) also expressed TBX19 (red), a specific transcriptional regulator of corticotroph lineage. (**j**,**k**) Electron micrograph of hESC-derived corticotrophs on day 88. Numerous secretory granules were seen close to the cell membrane. The boxed region in **j** is magnified in **k**. (**l**–**p**) Non-corticotroph differentiation. (**l**–**n**) Immunostaining of DX-treated aggregates for GH (**l**), PRL (**m**) and TSH (**n**; green). (**o**,**p**) LH and FSH immunostaining of DAPT-treated aggregates on day 82. Scale bars, 500 μm (**a**,**b**); 50 μm (**c**–**g**,**l**–**p**); 20 μm (**h**,**i**); 2 μm (**j**); 1 μm (**k**).

We next sought to determine whether pituitary placode tissues could differentiate into mature pituitary hormone-producing cells. First, we found that oral ectodermal tissues (outer layer) that initially did not form LHX3^+^ pouch-like structures later generated LHX3^+^ thick epithelia (Supplementary Fig. 3a). Since these non-pouch-forming LHX3^+^ portions were also positive for early pituitary markers (PITX1, and Islet1/2; Supplementary Fig. 3b,c), we examined whether both the LHX3^+^ non-pouch-like (Fig. 2a,b) and pouch-like (Fig. 2c) structures could generate pituitary hormone-producing cells. On days 67–70 (Supplementary Fig. 3d), both pouch-like (Fig. 2c) and non-pouch-like (Fig. 2d–f) thickened PITX1^+^ epithelia were observed. In both cases, ACTH^+^ corticotrophs were present (12.1±1.4% of PITX1^+^ cells; mean±s.e.m., *n*=4; see Methods; Fig. 2c,f and Supplementary Fig. 3e–k). These ACTH^+^ cells were Ki67^−^ (Fig. 2g) and LHX3^−^ (Fig. 2h), suggestive of postmitotic cells. The transcription factor *TBX19* (also known as *TPIT*), which is required for the corticotroph lineage^25^, was co-expressed with ACTH (Fig. 2i and Supplementary Fig. 3l). Furthermore, electron microscopy revealed that secretory granules were stored in the cytoplasm of these cells (Fig. 2j,k; arrowheads) and corticotropin-releasing hormone (CRH) receptors were expressed on the ACTH^+^ cells (Supplementary Fig. 3m,n; arrow), indicating that these corticotrophs were morphologically mature.

Previous studies using fetal rats have suggested that glucocorticoids themselves promote the development of somatotrophs^26^. We therefore treated hESC-derived pituitary tissues with the glucocorticoid dexamethasone (DX) during days 72–84 (Supplementary Fig. 3o), resulting in the appearance of GH^+^ cells (8.9±0.6% of PITX1^+^ cells; mean±s.e.m., *n*=4; see Methods; Fig. 2l and Supplementary Fig. 3p) that expressed their lineage marker POU1F1 (previously termed PIT1; Supplementary Fig. 3q). Furthermore, prolactin (PRL)^+^ cells and thyroid-stimulating hormone (TSH)^+^ cells (other POU1F1^+^ lineages^1^,^2^,^4^,^5^,^27^; Supplementary Fig. 3l) appeared, although these cells were comparatively rare (PRL^+^ cells <2% and TSH^+^ cells <1% of PITX1^+^ cells; Fig. 2m,n and Supplementary Fig. 3r).

In hESC culture, the Notch inhibitor (2S)-N-[N-(3,5-difluorophenacetyl)-L-alanyl]-2-phenylglycine tert-butylester (DAPT) is reported to induce the gonadotroph lineage^3^. We wondered, therefore, whether DAPT could be utilized *in vitro* to promote the differentiation of gonadotrophs. We found that 10-μM DAPT treatment during days 72–82 facilitated the appearance of luteinizing hormone (LH)^+^ cells and follicle-stimulating hormone (FSH)^+^ cells (Fig. 2o,p), and that the majority of the gonadotropic cells co-expressed LH and FSH, as seen *in vivo*^28^ (Supplementary Fig. 3s–u). Together, these findings demonstrated that 3D culture hESC-derived anterior pituitary progenitors are capable of generating multiple endocrine lineages. Two hESC lines (KhES-1 and KhES-3) were induced into anterior pituitary hormone-producing cells using the same protocol (Supplementary Fig. 3v,w).

**Figure 3:**
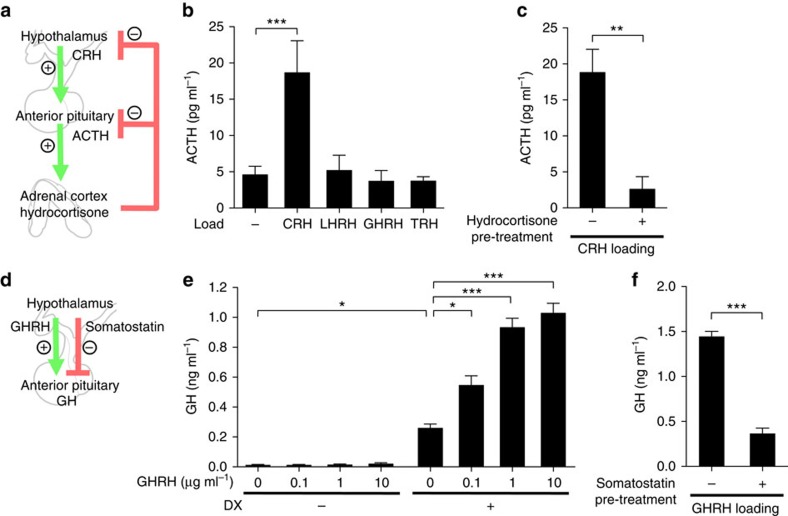
*In vitro* functionality of hESC-derived hormone-producing tissues. (**a**) Schematic of the hypothalamic–pituitary–adrenal axis. (**b**) CRH efficiently induced ACTH secretion (*n*=3 experiments). LHRH, luteinizing hormone-releasing hormone; TRH, thyrotropin-releasing hormone. (**c**) Pre-treatment with hydrocortisone suppressed the CRH-stimulated ACTH secretion from aggregates (*n*=5). (**d**) Schematic diagram of GH secretion *in vivo*. (**e**) DX treatment made aggregates release substantial amounts of GH in response to GHRH (*n*=3). (**f**) GH secretion was negatively regulated by somatostatin pre-treatment (*n*=3; 100 ng ml^−1^ for 90 min). Error bars represent s.e.m. *P<0.05, **P<0.01, ***P<0.001 using one-way ANOVA with Dunnett's test (**b**; versus control, **e**; versus DX (+)/GHRH (−) group), Student's *t*-test (**c**,**f**).

To address the functionality of the hESC *in vitro*-generated pituitary tissues, we first focused on corticotrophs, which were the cell type most robustly generated in our culture conditions. Corticotrophs release ACTH in response to CRH *in vivo* (Fig. 3a), and thus to test whether *in vitro* corticotrophs respond to CRH in a similar manner, we stimulated long-term cultured corticotrophs (days 83–84; Supplementary Fig. 3d) with CRH (Supplementary Fig. 4a) and measured ACTH release using enzyme-linked immunosorbent assay (ELISA) assays. These experiments showed that corticotrophs released a significant amount of ACTH when exposed to CRH (Supplementary Fig. 4b), but not by other hypothalamic releasing hormones (Fig. 3b). *In vivo*, CRH-induced ACTH release is suppressed by glucocorticoids, which are downstream in the pituitary–adrenal regulatory system^29^ (Fig. 3a) and act as feedback controls for hormonal homeostasis. Consistently, we found that, when hESC-derived corticotrophs were pre-treated with the glucocorticoid hydrocortisone for 3 h and then treated with CRH, the ACTH release was substantially suppressed (Fig. 3c).

We next examined the *in vitro* GH-secretion ability of induced somatotrophs (Supplementary Fig. 4c). GH secretion is stimulated by GH-releasing hormone (GHRH) *in vivo* (Fig. 3d). Although non-DX-treated aggregates secreted little GH regardless of GHRH loading, somatotrophs induced by DX treatment (Supplementary Fig. 3o) released substantial amounts of GH after GHRH stimulation (Fig. 3e). The basal level of GH secretion was also higher than non-DX-treated aggregates (Fig. 3e), suggesting the ability of DX to promote GH secretion without GHRH treatment *in vitro*^30^,^31^. When cultured in a hyperoxic (40% oxygen) condition from day 18, GH was more highly expressed than in a normoxic (20% oxygen) environment (Supplementary Fig. 4d–f). Furthermore, we found that GH secretion levels were downregulated by the somatostatin pre-treatment of the aggregates, just as seen *in vivo* (Fig. 3d,f). Together, these experiments demonstrated that *in vitro* hESC-derived pituitary tissues possess hormone-secretory behaviour that functions in line with known *in vivo* positive and negative regulatory signals.

### Therapeutic effects of transplantation to hypopituitary mice

We next investigated the *in vivo* functionality of hESC-derived pituitary tissues using hypophysectomized severe combined immunodeficient (SCID) mice. It was known that this type of pituitary resection is lethal within weeks in mice, mainly because of insufficiency of adrenal cortex functions caused by the lack of ACTH^9^. We wondered whether our *in vitro*-generated ACTH-producing cells, which were the most efficient type induced in our culture method, would be able to rescue the phenotypes of the hypophysectomized SCID mice following transplantation.

**Figure 4:**
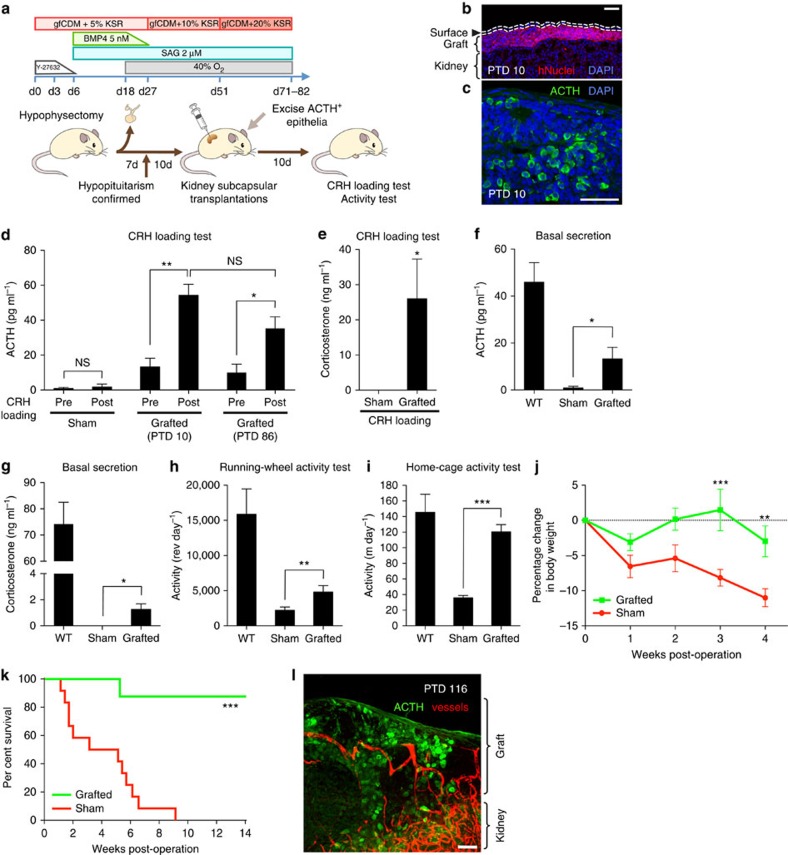
Rescue of hypopituitary mice by transplantation of hESC-derived corticotrophs. (**a**) Schematic of transplantation procedures. (**b,c**) Immunostaining of grafted tissue (post-transplant day 10) for human nuclei (**b**) and ACTH (**c**), both of which were found under the renal capsule. PTD, post-transplant day. (**d**) Blood-ACTH levels in sham-operated and grafted mice before and after CRH loading (PTD 10 and 86; *n*=3; that is, three mice each transplanted with the aggregates of three differentiation cultures). Sham, subcapsular saline injection. n.s., not significant. (**e**) Blood corticosterone levels on CRH loading (sampled 1 h after loading; PTD 10). Sham, *n*=4. Grafted, *n*=5. (**f**) Basal blood level (without CRH loading) of ACTH 10 days after transplantation. WT, wild type. Sham, *n*=5. Grafted, *n*=4. WT, *n*=3. (**g**) Basal blood level of corticosterone on PTD 10. Sham, *n*=5. Grafted, *n*=4. WT, *n*=6. (**h**) Spontaneous locomotor activity measured for 24 h by running wheels on PTD 10. Sham, *n*=8. Grafted, *n*=7. WT, *n*=4. (**i**) Home-cage activity (moving distance) measured by the infrared monitoring system (PTD 10). Sham, *n*=8. Grafted, *n*=7. WT, *n*=4. (**j**) Percentage change in body weight of grafted and sham-operated mice. Sham, *n*=6. Grafted, *n*=6. (**k**) Improved survival of transplanted hypopituitary mice. Sham, *n*=12. Grafted, *n*=8. (**l**) Immunostaining of PTD-116-grafted tissue for ACTH (green), and TRITC-gelatin-perfused blood vessels (red) of the host (Supplementary Movie 2). Scale bars, 50 μm (**b**,**c**,**l**). Error bars represent s.e.m. *P<0.05, **P<0.01, ***P<0.001 using paired *t*-test (**d**), Mann–Whitney test (**e**–**i**), two-way ANOVA (**j**) and log-rank test (**k**). All the *n*-values of transplantation experiments means; ‘*n*' mice each transplanted with the aggregates of ‘*n*' differentiation cultures.

After the hypopituitarism of host mice was confirmed by the depletion of ACTH in peripheral blood, we transplanted aggregate-derived pituitary placodes with ACTH^+^ cells subcapsularly into the kidneys of hypophysectomized mice (Fig. 4a and Supplementary Fig. 4g). Ten days after transplantation, the hESC-derived grafts formed thin subcapsular layers (Fig. 4b and Supplementary Fig. 4h) and contained ACTH^+^ cells (Fig. 4c). By CRH-loading tests (10 days after operation), we found that the grafted group exhibited a substantial induction of ACTH release compared with the sham-operated group (Fig. 4d). Plasma corticosterone levels were also significantly higher in the grafted group (Fig. 4e), and basal levels of ACTH and corticosterone were also increased (Fig. 4f,g). (Before these experiments, we demonstrated that human ACTH had the ability to induce a substantial elevation of blood ACTH levels in wild-type SCID mice; Supplementary Fig. 4i.) These results indicated that ACTH from the grafts sufficiently stimulated the host adrenal glands and were functional within the host's hormonal system.

Hypophysectomy-induced adrenal insufficiency results in low physical activity levels in mice^32^. When we compared the grafted and control groups using running-wheel and home-cage activity tests, we found that the grafted group showed significantly higher activity levels than the sham-operated group (Fig. 4h,i and Supplementary Movie 1), demonstrating that hESC-derived pituitary tissues have the ability to recover the physical activities of the host. Since these effects were seen without CRH loading, the partial elevation of the basal ACTH levels (Fig. 4f) was sufficient to increase the vitality of hypophysectomized mice.

Finally, we evaluated the long-term effects of corticotroph transplantation on host body weight and survival. The control hypophysectomized mice gradually lost ∼10% of their body weight over 4 weeks (Fig. 4j). In contrast, the grafted mice kept body weights that were close to pre-transplantation levels (Fig. 4j). In addition, the grafted group showed longer survival than the control group (Fig. 4k; half of the control hypophysectomized mice died by 5 weeks after operation and the majority of the grafted mice survived to 14 weeks). This survival capacity was probably due to the retained functionality of the pituitary grafts because, in grafted mice surviving 12 weeks after transplantation, the secretory capacity of grafted ACTH^+^ cells was found to be at a similar level as after the initial transplantation (Fig. 4d). In addition, we found that the ACTH-producing cells still existed in kidney subcapsular lesions 16 weeks after grafting and had formed new vasculature within the graft (Fig. 4l and Supplementary Movie 2), and no tumorigenesis was observed during the follow-up period. These data show that pituitary grafts integrate into their host mice, allowing long-term survival and increased vitality and body weight.

## Discussion

In summary, this study is the first to show both the efficient stem-cell culture for human pituitary endocrine cells with precise hormonal responses and *in vivo* rescue of hypopituitarism by transplantation. Our present study is based on the premises that human pituitary development resembles mouse pituitary development. Our previous mESC protocol^11^ and the present hESC protocol are similar in that both involve 3D culture to recapitulate the interaction between hypothalamus and oral ectoderm. However, in the mESC culture, hypothalamic NE and oral ectoderm were co-induced by endogenous BMP4 (ref. 11); however, in this hESC culture, exogenous BMP4 (5 nM final concentration) was needed to induce them. Another intriguing difference between mESC and hESC cultures for pituitary differentiation was the formation of Rathke's pouch-like vesicles. In our previous study using mESC culture^11^, pituitary placode tissues efficiently invaginated to form pouches and generated hormone-producing cells, while the rest of the oral ectoderm stayed on the surface and formed a thin epithelium that did not express pituitary-specific markers even at a later stage^11^. In contrast, hESC-cultured tissues formed LHX3^+^ pouches (especially in the presence of FGF2), the majority of which did not invaginate and instead remained on the epithelial surface. These tissues became LHX3^+^ pituitary placode after long-term culture and were able to generate hormone-producing cells. Thus, the morphological formation of pouch structures is neither required for terminal specification nor for function of hESC-generated hormonal cells. Furthermore, the effect of the Notch inhibitor DAPT is also different in mESC versus hESC cultures. It is interesting that Notch inhibition is needed for corticotrophs to develop from mESC culture^11^, whereas in hESC culture, Notch inhibition leads to gonadotrophs, not corticotrophs. Although Dincer's protocol and our hESC protocol basically differ in two-dimensional versus 3D culture, Dincer's protocol also requires the Notch inhibitor to induce gonadotrophs^3^, suggesting that the effect of DAPT is common in human pluripotent stem cells. Furthermore, our 3D culture systems seem to recapitulate the human developmental process, at least in part, suggesting that they could be used as a model of human embryology and disease pathology.

Using our culture method, we succeeded in generating mature pituitary endocrine cells such as corticotrophs, which secreted a substantial amount of ACTH in response to CRH. Notably, this release was suppressed by a negative feedback mechanism by a downstream hormone glucocorticoid, as seen *in vivo*. These findings show that our hESC culture produced human corticotrophs with proper responses to upstream and downstream signals, a promising sign that such tissue could be used in future replacement therapies. In addition to corticotrophs, we also created somatotrophs and confirmed their faithful GH secretion in response to GHRH and somatostatin *in vitro*, a feat that was not achieved in previous studies using mESCs^11^. Dincer *et al.*^3^ reported the generation of human somatotrophs *in vitro*, but GH secretion was demonstrated only *in vivo*. Moreover, GH secretion tests using GHRH or somatostatin were not performed^3^. Thus, our report is the first to outline the *in vitro* generation of fully functional GH-secreting somatotrophs from pluripotent stem cells, although we do not confirm their functionality *in vivo*.

Interestingly, we found that the differentiation programmes delineating the maturation of GH- and ACTH-producing cells were greatly influenced by the presence or absence of DX treatment, respectively. Thus, DX is a useful reagent that can facilitate the selective *in vitro* differentiation of somatotrophs versus corticotrophs. Future studies will aim to explore the mechanisms behind DX-induced somatotroph differentiation.

Finally, we performed transplantation studies in hypophysectomized mice, whereas previous studies^3^ were performed in normal (immunodeficient) mice and rats. By using hypopituitary mice, we found that induced corticotrophs can secrete ACTH in response to its natural releasing factor CRH. These levels were sufficient to induce glucocorticoid release. Moreover, the daily activities of the host mice were recovered and their survival was clearly elongated. Thus, our study goes beyond previous work^3^ to show not only proper regulation of the *in vitro*-generated endocrine cells by upstream and downstream hormones but also their therapeutic ability when transplanted into hypopituitary hosts. This represents an important step towards the application of pluripotent stem cells in the treatment of endocrine disorders. For the clinical application, the differentiation efficiency needs to be enhanced.

Our laboratory is planning to apply this method to a human-induced pluripotent stem cell system in the near future. In general, human-induced pluripotent stem cells have fewer ethical problems, but further examinations are needed for certification of its safety. Already, clinical trials of the human ES/iPS cell-derived retinal epithelium are underway^33^,^34^. We also endeavour to develop xeno-free culture systems for clinical application.

## Methods

### Maintenance and differentiation culture of hESCs

hESCs (KhES-1 and KhES-3 (RIKEN BioResource Center, Cell Number: HES0001 and HES0003, respectively)) were used in accordance with the hESC research guidelines of the Japanese government. Undifferentiated hESCs were maintained on a feeder layer of mouse embryonic fibroblasts inactivated by mitomycin C treatment in DMEM/F-12 (Sigma) supplemented with 20% (vol/vol) KSR (Invitrogen), 2 mM glutamine, 0.1 mM nonessential amino acids (Invitrogen), 5 ng ml^−1^ recombinant human basic FGF (Wako) and 0.1 mM 2-mercaptoethanol under 2% CO_2_. For passaging, hESC colonies were detached and recovered *en bloc* from culture dishes by treating with 0.25% (wt/vol) trypsin and 1 mg ml^−1^ collagenase IV in PBS containing 20% (vol/vol) KSR and 1 mM CaCl_2_ at 37 °C for 10 min. The detached hESC clumps were broken into smaller pieces by pipetting. The passages were performed at a 1:6 split ratio every fourth day.

For SFEBq culture, hESCs were dissociated to single cells in TrypLE Express (Invitrogen) containing 0.05 mg ml^−1^ DNase I (Roche) and 10 μM Y-27632. They were quickly aggregated using low-cell-adhesion 96-well plates with V-bottomed conical wells (Sumilon PrimeSurface plate; Sumitomo Bakelite) in differentiation medium (5,000 cells per well, 100 μl) containing 20 μM Y-27632. The differentiation medium (gfCDM) was supplemented with 5% KSR. The gfCDM comprises Iscove's modified Dulbecco's medium/Ham's F12 1:1, 1% chemically defined lipid concentrate, monothioglycerol (450 μM) and 5 mg ml^−1^ purified bovine serum albumin (>99% purified by crystallization; Sigma). Defining the day on which the SFEBq culture was started as day 0, 100 μl per well gfCDM was added to each well on day 3. From days 6 to 27, the medium is half renewed every 3 days. SAG (Enzo Life Sciences) and recombinant human BMP4 (R&D) were added to culture to reach 2 μM and 5 nM, respectively, from day 6. BMP4 concentrations were diluted by half-volume changes with BMP4-free medium every third day from day 18. For promoting Rathke's pouch formation, 20 ng ml^−1^ recombinant human FGF2 (R&D) was added to culture media from days 15 to 27, even though this FGF2 treatment caused no substantial changes in the differentiation of hormone-producing cells. From day 18, aggregates were cultured under the high-O_2_ condition (40%). After culturing in a 96-well plate for 27 days, aggregates were transferred to a 10-cm Petri dish for suspension culture in gfCDM supplemented with 10% KSR and 2 μM SAG on day 27 exactly. From day 30, a full medium change was performed every third day. The concentration of KSR was increased (final 20% (vol/vol)) from day 50. SAG (2 μM) was needed throughout the culture (from day 6) because we found that the aggregates had few pituitary placodes after omission of SAG from day 30.

For inhibition studies, PD173074 (Millipore), SU5402 (Millipore), HPI-1 (Sigma) and GANT61 (Wako) were obtained commercially.

### Immunohistochemistry

Immunohistochemistry of frozen sections was performed with primary antibodies described below. The antibodies were used at the following dilutions: FOXG1 (rabbit, 1:3,000)^35^, GFP (rabbit, 1:500; MBL; rat, 1:500, Nacalai Tesque), NKX2.1 (mouse, 1:1,000; Millipore), pan-cytokeratin (mouse, 1:100; Sigma), aPKC (rabbit, 1:100; Santa Cruz), ECAD (rat, 1:50; TaKaRa), NCAD (mouse, 1:1,000; BD), TUJ (rabbit, 1:500; Covance), RX (guinea pig, 1:3,000), LHX3 (rabbit, 1:3,000), PITX1 (guinea pig, 1:2,000), ISL1/2 (mouse, 1:50; DSHB), CHX10 (goat, 1:100; Santa Cruz), ACTH (mouse, 1:200, Fitzgerald; mouse, 1:50, Dako), GH (rabbit, 1:800; Dako), PRL (rabbit, 1:300; Dako), TSH (mouse, 1:50; Dako), LH (mouse, 1:50, Dako; goat, 1:160, Santa Cruz), FSH (mouse, 1:50; Dako), TBX19 (guinea pig, 1:2,000), POU1F1 (guinea pig, 1:2,000), Ki67 (rabbit, 1:1,500; Leica), PC1/3 (rabbit, 1:100; Novus Biologicals), PC2 (rabbit, 1:500; Proteintech) and CRH-R (goat, 1:100; Santa Cruz), human nuclei (mouse, 1:25; Millipore). Polyclonal antisera against RX were generated in guinea pigs by immunizing with recombinant glutathione *S*-transferase (GST)-tagged N-terminal 90 residues of human RX (FTKDDGILGTFPAERGARGAKERDRRLGARPACPKAPEEGSEPSPPPAPAPAPEYEAPRPYCPKEPWEARPSPGLPVGPATGEAKLSEEE) that were purified using affinity chromatography or gel extraction after SDS–PAGE. To enhance the specificity of antigen–antibody reaction, these antisera were passed through an absorber GST protein–sepharose column and subsequently affinity-purified using MBP-tagged N-terminal 90 residues of RX described above. The antiserum against LHX3 was raised in rabbits against a synthetic peptide (C-PSSDLSTGSSGGYPDFPASPASWLDEVDHAQF; residues 366–397). The antiserum against PITX1 was raised in guinea pigs against a synthetic peptide (DPREPLENSASESSDTELPEKERGGEPKGPEDSGAGGTG-C; residues 38–77). The antiserum against TBX19 was raised in guinea pigs against synthetic peptides (C-KIKYNPFAKAFLDAKERNHL; residues 206–225 and C-LRDVPEAISESQHVTY; residues 225–240). The antiserum against POU1F1 was raised in guinea pigs against synthetic peptides (C-LAEDPTAADFKQELRRKSKL; residues 98–117 and C-LYNEKVGANERKRKRRTTI; residues 203–221). The validity of these custom antibodies was confirmed by positive staining control (Supplementary Fig. 5a–f). Counter nuclear staining was performed with 4,6-diamidino-2-phenylindole (Nacalai Tesque).

### Quantitative PCR

Quantitative PCR (qPCR) was performed with eight aggregates per sample using the 7500 Fast Real Time PCR System (Applied Biosystems) and the data were normalized to the ACTB expression. Primers used were as follows: ACTB, forward 5′-TCCCTGGAGAAGAGCTACG-3′, reverse 5′-GTAGTTTCGTGGATGCCACA-3′; PITX1, forward 5′-TCCACCAAGAGCTTCACCTT-3′, reverse 5′-CGGTGAGGTTGTTGATGTTG-3′; LHX3, forward 5′-GGCTGGCCTGTGTGTAAGTC-3′, reverse 5′-CATTCACAGAACCAATAGGTAGCTC-3′; GLI1, forward 5′-GGGATGATCCCACATCCTCAGTC-3′, reverse 5′-CTGGAGCAGCCCCCCCAGT-3′; GLI2, forward 5′-TGGCCGCTTCAGATGACAGATGTTG-3′, reverse 5′-CGTTAGCCGAATGTCAGCCGTGAAG-3′; GLI3, forward 5′-GGCCATCCACATGGAATATC-3′, reverse 5′-TGAAGAGCTACGGGAAT-3′; FGF8, forward 5′-AGCAGAGTTCGAGTCCGAGGAG-3′, reverse 5′-CAGCGCTGTGTAGTTGTTCTCCA-3′; FGF10, forward 5′-CTGGAGATAACATCAGTAGAAATCG-3′, reverse 5′-GAGCAGAGGTGTTTTTCCTTCGT-3′.

### Electron microscopy

Aggregates were fixed with 2% fresh formaldehyde and 2.5% glutaraldehyde in 0.1 M sodium cacodylate buffer (pH 7.4) for 2 h at room temperature. After washing with 0.1 M cacodylate buffer (pH 7.4) three times (5 min each), they were postfixed with ice-cold 1% OsO_4_ in the same buffer for 2 h. The aggregates were rinsed with distilled water, stained with 0.5% aqueous uranyl acetate for 2 h or overnight at room temperature, dehydrated with ethanol and propylene oxide and embedded in Poly/Bed 812 (Polyscience)^36^. Ultrathin sections were cut, doubly stained with uranyl acetate and Reynold's lead citrate and viewed with a JEM 1010 transmission electron microscope (JEOL) at an accelerating voltage of 100 kV.

### Statistical analyses

All data were analysed using the Prism 5 software (GraphPad). For quantification of hormone-positive cell numbers, at least 2,000 cells, which expressed PITX1, were counted in eight different fields from four biologically independent experiments.

### CRH and GHRH loading test *in vitro*

Sixteen aggregates were collected on day 80 in a 1.5-ml Eppendorf tube, rinsed with HBSS and pre-incubated in 250 μl HBSS at 37 °C for 10 min. Human CRH (1 μg ml^−1^) was then added. The supernatant was collected after 10-min incubation at 37 °C and subjected to ELISA using the ACTH ELISA kit (MD Bioproducts).

Thirty aggregates were collected on day 84, rinsed with differentiation medium and pre-incubated in 750 μl medium at 37 °C for 30 min. Human GHRH was then added to the given concentrations. The supernatant was collected after 30-min incubation at 37 °C and subjected to ELISA using the GH ELISA kit (Roche).

### Hydrocortisone-induced ACTH suppression test

Sixteen aggregates were pre-treated with human hydrocortisone (20 μg ml^−1^) for 3 h on day 80. Then, they were collected in a 1.5-ml Eppendorf tube, rinsed with HBSS and CRH (100 ng ml^−1^ final concentration) was added to 250 μl HBSS. The supernatant was collected after 10-min incubation at 37 °C and subjected to ELISA using the ACTH ELISA kit (MD Bioproducts).

### Somatostatin-induced GH suppression test

We pre-treated 18 aggregates with human somatostatin (100 ng ml^−1^) for 90 min on day 98. They were collected in a 1.5-ml Eppendorf tube, rinsed with gfCDM+20% KSR medium and human GHRH (100 ng ml^−1^ final concentration) was added to 750 μl medium. The supernatant was collected after 30-min incubation at 37 °C and subjected to ELISA using the GH ELISA kit (Roche).

### Transplantation of hESC-derived pituitary tissues

All animal experiments were performed in accordance with the institutional (RIKEN) guidelines for animal studies. Surgical hypophysectomy of SCID mice (8-week-old males) was performed using a transaural approach^37^. Briefly, mice were anaesthetized with intraperitoneal (i.p.) injection of pentobarbital (40 mg kg^−1^), and the pituitary tissues were aspirated from the sella turcica using a needle (KN-390 needle, Natsume Seisakusyo) set to a 1-ml syringe containing 0.2 ml saline, following its perforation via the auditory meatus. The mice were kept in cages under conditions that minimize stress. Seven days later (9-week-old), CRH-stimulated (2 μg kg^−1^, i.p.) blood levels of ACTH were analysed to confirm the hypopituitarism status. Ten days later, the 10-week-old hypopituitary mice were anaesthetised and injected with hESC-derived pituitary placodes (in 100 μl normal saline) into the left kidney under the capsule using a 21-G needle syringe. The pituitary placodes, identified by their translucent and thin epithelia relative to surrounding NE, were excised from the surface of aggregates (10 aggregates per host mouse). The kidney was exposed by skin/muscle/peritoneum incision via the dorsolateral approach. Before the operation, the mice were injected with DX (0.2 mg) and ampicillin (2 mg) intramuscularly. This DX supplementation was essential for good post-operational survival of hypopituitary mice, which are very weak against physical stress. Ten days later (12-week-old), the grafted mice were subjected to a CRH-loading test (2 μg kg^−1^, i.p.) and blood sampling was performed 1 h after the human CRH injection. The ‘basal' secretion levels of ACTH and corticosterone (without CRH loading) were examined at 20:00 in a stress-free environment. The blood ACTH levels of mice were measured using an ACTH ELISA kit (MD Bioproducts), which crossreacts with human and mouse ACTH. The spontaneous locomotor tests (12-week-old male mice) were performed under a stress-free condition in the mice's home cages using a running-wheel device (ENV-044; MedAssociates) and an infrared 24-h monitoring system (MDC-W02; BrainScienceIdea).

### Vascular imaging using TRITC–gelatin conjugate

Tetramethylrhodamine-5-(and 6)-isothiocyanate (TRITC; Thermo) was conjugated to gelatin (Sigma-Aldrich). TRITC (10 mg) was dissolved in 1 ml dimethylsulphoxide (Sigma-Aldrich) at pH 11. TRITC solution and 5% (wt/vol) gelatin solution at pH 11 were mixed for conjugation at 37 °C overnight. The unconjugated TRITC was removed with a NAP-25 column (GE Healthcare UK Ltd.). Animals were thoracotomized under deep anaesthesia and flushed using 20 ml saline via left ventricular. After flushing, 6 ml of filtrated TRITC-conjugated gelatin was perfused continuously and the kidney was fixed with 8% formalin at ice temperature.

## Additional information

**How to cite this article:** Ozone, C. *et al.* Functional anterior pituitary generated in self-organizing culture of human embryonic stem cells. *Nat. Commun.* 7:10351 doi: 10.1038/ncomms10351 (2016).

## Supplementary Material

Supplementary Information Supplementary Figures 1-5

Supplementary Movie 1 Improved locomotor activity in a pituitary-resected mouse receiving hESC-derived corticotrophs. Spontaneous locomotor activity measured by the running-wheel test (10 days after transplantation). Sham-operated; subcapsular saline injection. The hypophysectomized mouse with ACTH-producing aggregates was more active than the sham-operated mouse.

Supplementary Movie 2 3D imaging of grafted ACTH+ tissue under the renal capsule. Immunostaining of PTD-116 grafted tissue for ACTH (green) and TRITC-gelatin-perfused blood vessels (red) of the host. The ACTH+ cells existed in kidney subcapsular lesions even 16 weeks after transplantation and the formation of new blood vessels was observed within the graft.
