# Supplementary material for: Functional anterior pituitary generated in self-organizing culture of human embryonic stem cells
Source: Nat Commun. 2016 Jan 14;7:10351. doi: 10.1038/ncomms10351 (PMC4735598; doi:10.1038/ncomms10351)
Supplement: Supplementary Information — Supplementary Figures 1-5 [file ncomms10351-s1.pdf]

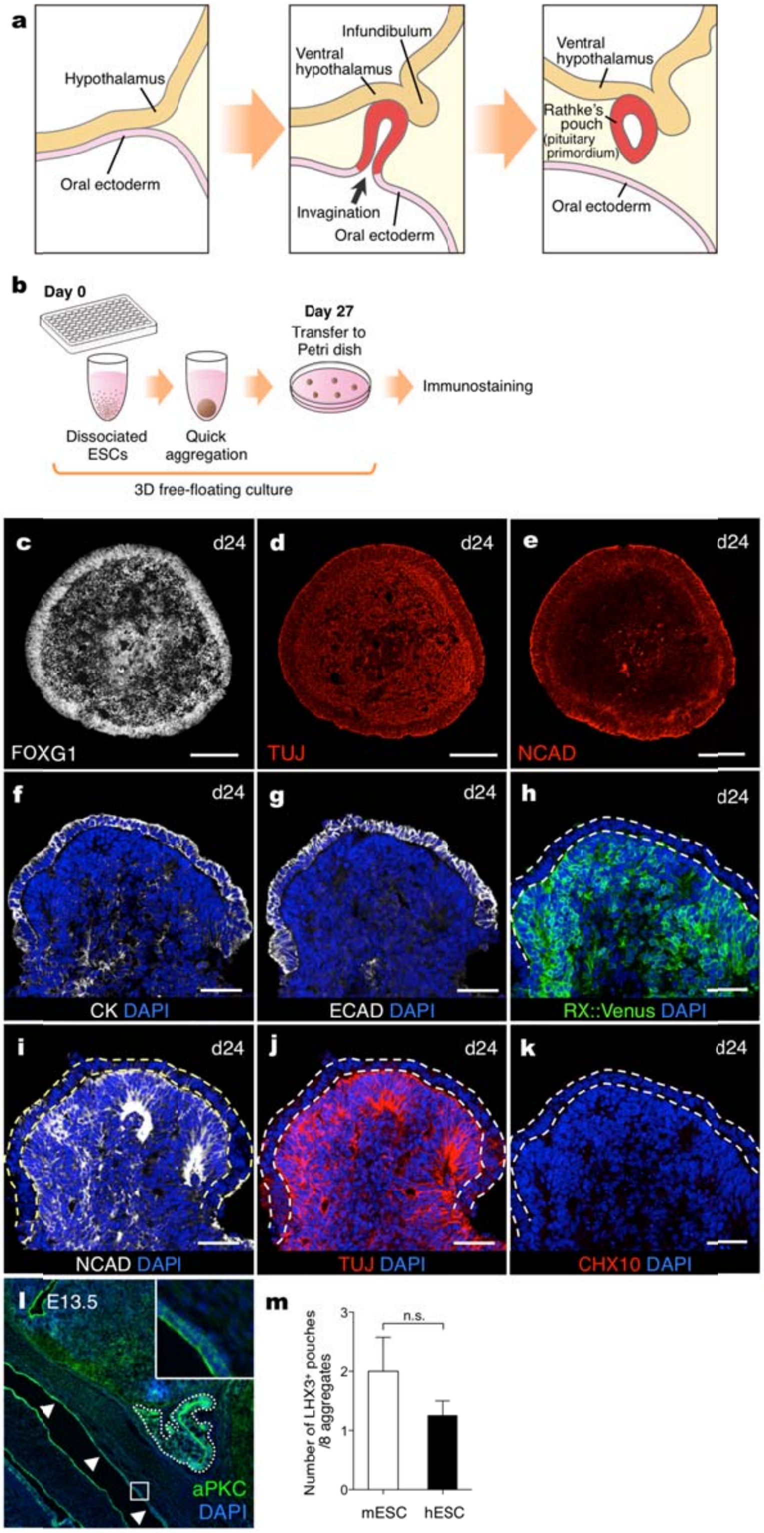
a
Hypothalamus
Oral ectoderm
Infundibulum
Ventral hypothalamus
Invagination
Oral ectoderm
Ventral hypothalamus
Rathke's pouch
(pituitary primordium)
Oral ectoderm
b
Day 0
Dissociated ESCs
Quick aggregation
Day 27
Transfer to Petri dish
Immunostaining
3D free-floating culture
c d24 FOXG1
d d24 TUJ
e d24 NCAD
f d24 CK DAPI
g d24 ECAD DAPI
h d24 RX::Venus DAPI
i d24 NCAD DAPI
j d24 TUJ DAPI
k d24 CHX10 DAPI
l E13.5
aPKC
DAPI
m
Number of LHX3+ pouches /8 aggregates
n.s.
mESC
hESC

Supplementary Figure 1 | Simultaneous generation of non-neural oral ectoderm and hypothalamic tissue in three-dimensional human embryonic stem cell (hESC) culture.

(**a**) Schematic diagram of mouse pituitary development, sagittal view. (**b**) Schematic diagram of serum-free floating culture of embryoid body-like aggregates with quick reaggregation (SFEBq) culture. (**c-e**) Immunostaining of a day-24 aggregate without smoothened agonist (SAG) treatment for FOXG1 (**c**), TUJ (**d**) and N-cadherin (NCAD; **e**). The aggregate without SAG co-expressed FOXG1, TUJ and NCAD, indicating that the telencephalic neuroectoderm (NE) was induced. c and d; the same section, c and e; serial sections. (**f**,**g**) Non-neural oral ectoderm (pan-Cytokeratin^{+}; **f**) also expressed E-cadherin (ECAD; white; **g**). (**h-k**) Induced *RX*::Venus^{+} tissue expressed the neural markers NCAD (**i**) and TUJ (**j**), but not the retinal marker CHX10 (**k**). (**l**) Atypical kinase C (aPKC) immunostaining of a sagittal section of the E13.5 mouse pituitary primordium demarcated by a white dotted line and oral ectoderm (arrowheads). The is a magnification of the white outlined area. (**m**) The number of Rathke's pouch-like structures (LHX3^{+}) of human versus mouse ESC aggregates (per 8 aggregates; hESC; *n* 4, mESC; *n* = 3 experiments). n.s., not significant. Error bars represent s.e.m. Student's *t*-test. Scale bars; 200 μm (**c-e**); 50 μm (**f-k**).

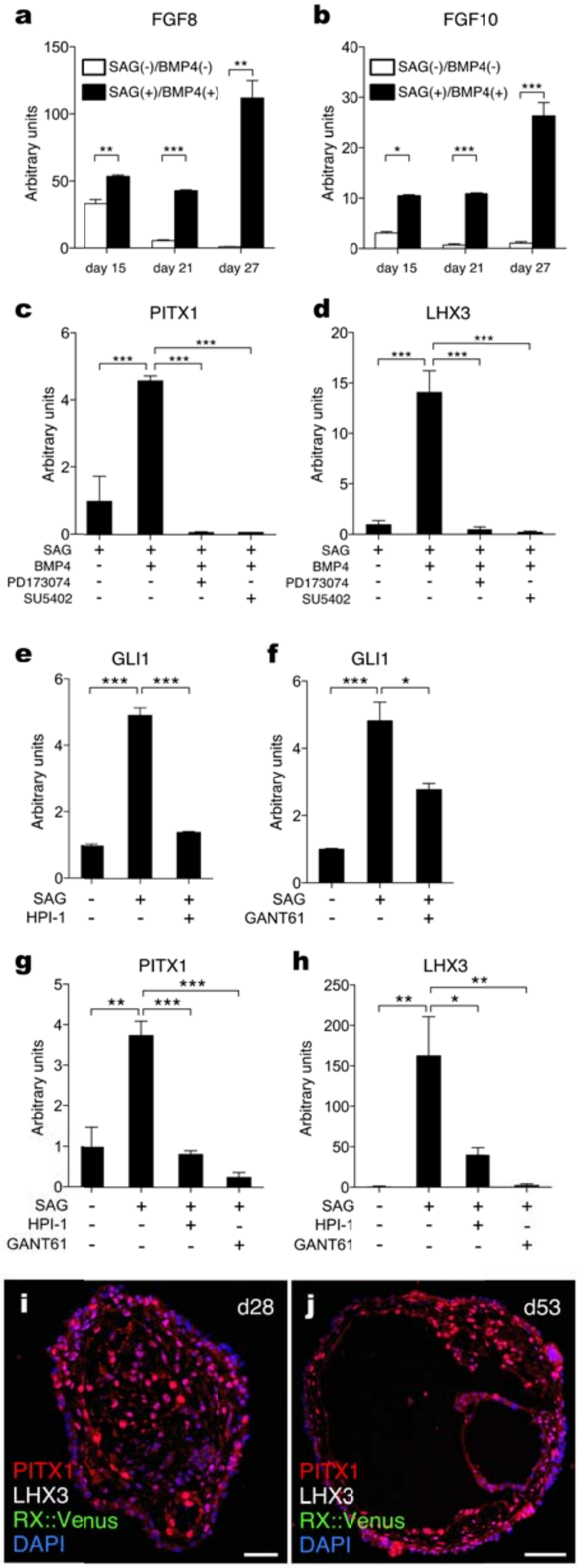

a
FGF8
SAG(-)/BMP4(-)
SAG(+)/BMP4(+)
Arbitrary units
day 15
day 21
day 27
b
FGF10
SAG(-)/BMP4(-)
SAG(+)/BMP4(+)
Arbitrary units
day 15
day 21
day 27
c
PITX1
d
LHX3
SAG
BMP4
PD173074
SU5402
e
GLI1
f
GLI1
SAG
HPI-1
GANT61
g
PITX1
h
LHX3
i
d28
j
d53
PITX1
LHX3
RX::Venus
DAPI

Supplementary Figure 2 | Roles of fibroblast growth factor (FGF) and hedgehog signals in the hESC culture for the differentiation of PITX1^{+} and LHX3^{+} epithelium.

(**a**,**b**) The endogenous expression of *FGF8* (**a**) and *FGF10* (**b**) during days 15-27 in the aggregates treated or untreated with SAG (2 μM) and bone morphogenetic protein 4 (BMP4, 5 nM; qPCR analysis; n = 3 experiments). (**c**,**d**) The aggregates treated with SAG (2 μM) and BMP4 (5 nM) expressed *PITX1* and *LHX3*, but FGF inhibitor treatment, PD173074 (10 μM) and SU5402 (0.5 μM) on days 15-27, decreased their expression on day 27 (qPCR; n = 3 experiments). (**e**,**f**) qPCR analysis for expression of *GLI1*. SFEBq culture on day 8. SAG treatment increased the *GLI1* expression, but treatment with GLI inhibitors (HPI-1 at 50 μM for 48 hours; GANT61 30 μM for 36 hours) decreased *GLI1* expression of the SAG-treated aggregates (n = 3 experiments). (**g**,**h**) Inhibition of *PITX1* (**g**) and *LHX3* (**h**) induction (on day 27) by treating hESC aggregates with the GLI inhibitors during days 6-27 (qPCR; n = 3). (**i**,**j**) PITX1^{+} oral ectoderm (red) alone did not express LHX3 (white). (**i**) on day 28. (**j**) on day 53. (These aggregates, which consisted of only oral ectoderm, were quite rare in our culture.) Error bars represent s.e.m. * $P < 0.05$, ** $P < 0.01$, *** $P < 0.001$ using one-way ANOVA with Bonferroni's test (**a-h**). Scale bars, 50 μm (**i**); 100 μm (**j**).

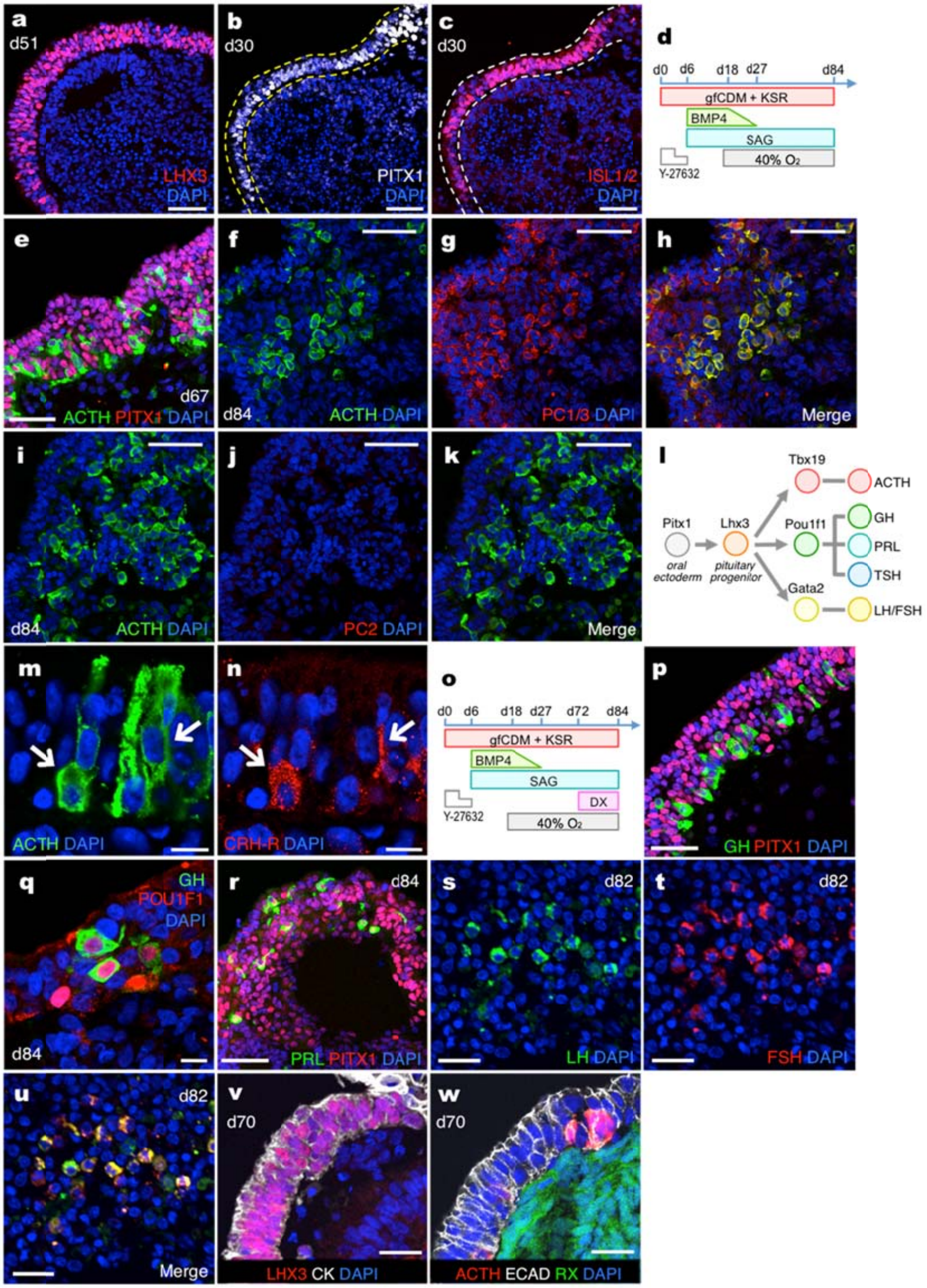

Supplementary Figure 3 | Generation of multiple endocrine lineages in 3D hESC culture.

(**a**) LHX3 immunostaining of day-51 aggregates. (**b**, **c**) PITX1^{+} surface ectoderm

(white; **b**) expressed pituitary progenitor marker ISL1/2 (red; **c**), on day 30. (**d**) Culture protocol for adrenocorticotropic hormone (ACTH)-producing cells. (**e**) Double-staining of a day-67 aggregate for ACTH (green) and PITX1 (red). (**f-k**) Induced $ACTH^+$ cells expressed the corticotroph marker PC1/3 (**f-h**) but not the melanotroph marker PC2 (**i-k**). (**l**) Schematic of pituitary lineage development *in vivo*. (**m**,**n**) $ACTH^+$ cells (green; **m**) expressed corticotropin releasing hormone receptors (CRH-R; **n**). (**o**) Culture protocol for growth hormone (GH)-secreting cells. DX, dexamethasone. (**p**) A human ESC aggregate immunostained for GH (green) and PITX1 (red). (**q**) Immunostaining of day-84 dexamethasone-treated aggregates for GH (green) and the lineage marker POU1F1 (red). (**r**) Prolactin (PRL; green) co-expressed with PITX1 (red) in hESC aggregates. (**s-u**) The majority of hESC-derived gonadotoropic cells co-expressed luteinizing hormone (LH; green) and follicle stimulating hormone (FSH; red). (**v**,**w**) Immunostaining of aggregates derived from a hESC line, KhES-3. (**v**) LHX3 (red) and pan-Cytokeratin (white). (**w**) ACTH (red), ECAD (white) and RX (green). In this report, all the hESC-aggregates' data except for Supplementary Fig. 3s,t was derived from the KhES-1 cell line. Scale bars, 50 μm (**a-c**,**e-k**,**p**,**r**); 10 μm (**m**,**n**,**q**); 20 μm (**s-w**).

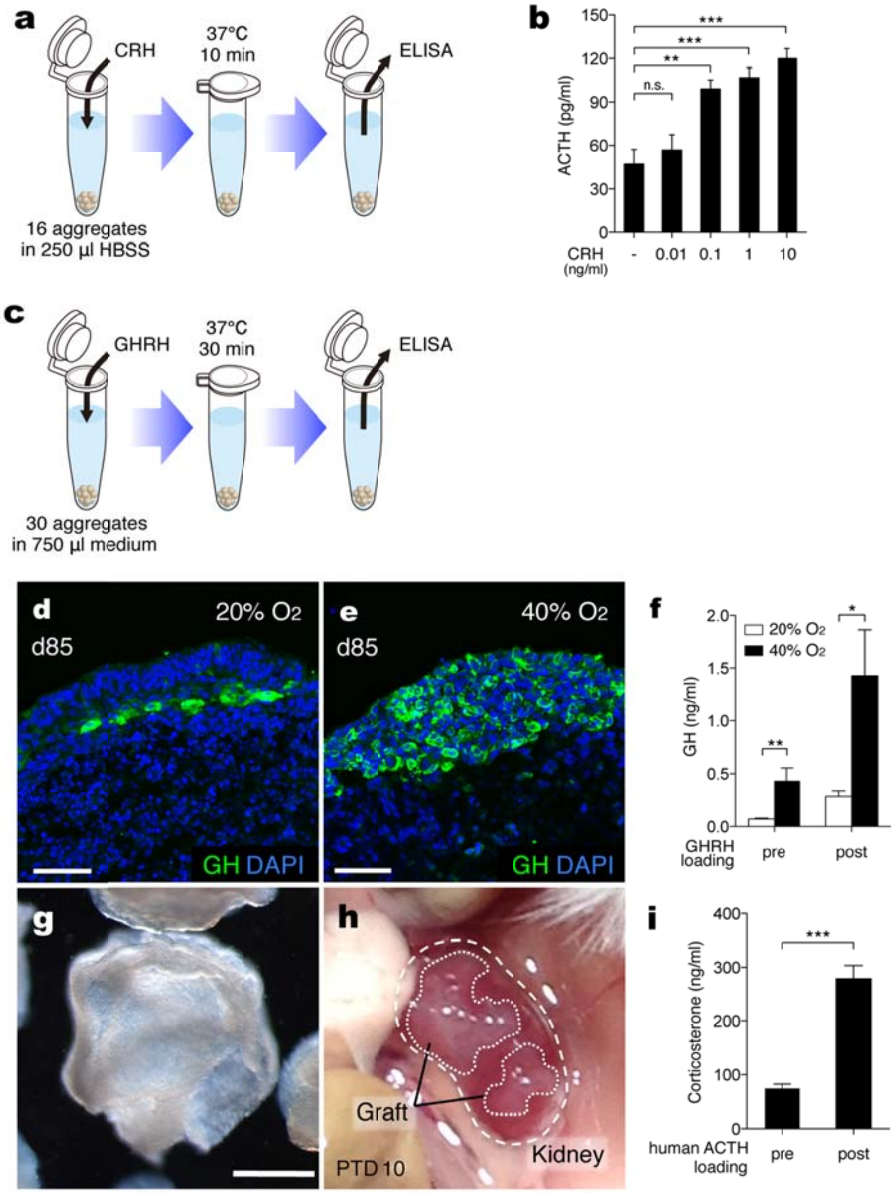

Supplementary Figure 4 | *In vitro* hormone secretion test and transplantation of hESC-derived corticotrophs.

(**a**) Schematic of *in vitro* CRH-loading test for ACTH secretion. (**b**) ACTH secretion from day-89 aggregates at different CRH doses (n = 3 experiments). (**c**) Schematic of growth hormone releasing hormone (GHRH)-loading test for GH secretion. (**d**, **e**) GH immunostaining of day-94 aggregates cultured in atmospheric (20% O_2; **d**) versus high-oxygen (40% O_2 from day 18; **e**) environment. (**f**) GHRH loading test showed that

the day-94 aggregates cultured in hyperoxic (40% O_2) condition secreted more GH versus control (n = 7). (**g**) Pituitary epithelial lesion excised from hESC aggregates, i.e. NE and debris were removed to reduce the graft volume. (**h**) The grafts 10 days after transplantation under the mouse renal capsule. (**i**) Increased blood corticosterone levels of wild-type SCID mice 60 min after intraperitoneal administration of human ACTH (5 ng/g; n = 6 experiments). Scale bars, 50 µm (**d**,**e**); 200 µm (**g**). Error bars represent s.e.m. * $P < 0.05$, ** $P < 0.01$, *** $P < 0.001$ using one-way ANOVA with Bonferroni's test (**b**) and Student's *t*-test (**f**,**i**).

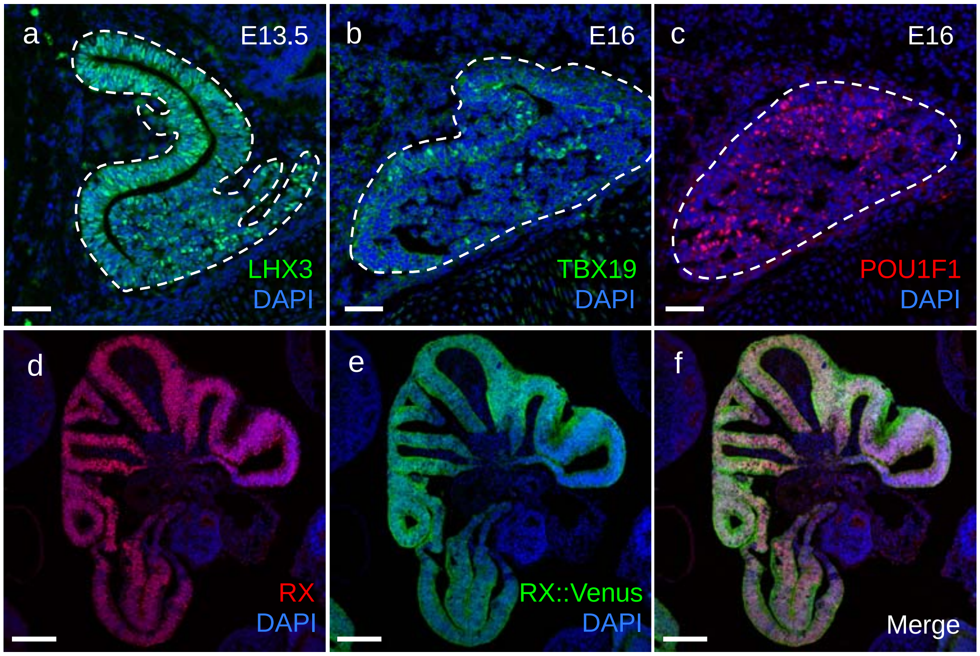

Supplementary Figure 5 | The specificity of antibodies.

(**a-c**) The anti-human LHX3 antibody was raised against a synthetic peptide whose amino acid sequence was nearly the same in human and mouse. The anti-human TBX19, and POU1F1 antibodies were raised against synthetic peptides whose amino acid sequence was the same in human and mouse. These antibodies (LHX3; **a**, TBX19; **b**, POU1F1; **c**) selectively recognized mouse embryonic adenohypophysis. (**d-f**) The specificity of anti-RX antibody was confirmed by immunostaining of *RX*::Venus hESC reporter line-derived neural retina. Scale bars, 50 µm (**a-c**); 200 µm (**d-f**).
